# COMPARING DATA-DRIVEN AND THEORY-DRIVEN APPROACHES TO CLASSIFY SOCIAL RELATIONSHIPS ACROSS THE LIFE COURSE

**DOI:** 10.1093/geroni/igac059.2623

**Published:** 2022-12-20

**Authors:** Elliot Friedman, Elizabeth Teas, Kristine Marceau

**Affiliations:** Purdue University, West Lafayette, Indiana, United States; Purdue University, Lafayette, Indiana, United States; Purdue University, West Lafayette, Indiana, United States

## Abstract

A life course perspective on social relationships highlights the salience of specific relationships at specific times in life, but analyses that account for life course trajectories in social relationships are rare. The present study explores the properties of two analytic approaches to classifying life course relationships: 1) a priori, or theoretical, classification strategies, and 2) latent profile analysis (LPA). Including multiple dimensions of social relationships at different time points across the life course, we determined whether theoretical or LPA profiles better predict later-life functional limitations, given that functional impairment is prevalent among middle-aged and older adults. We also assessed whether the two approaches are differentially associated with a broad set of covariates. Data were from three waves of the Midlife in the United States (MIDUS) study (n = 6,909). Relationship variables (parental affection, parental discipline, social support, social strain, and positive relations with others) were from wave 1. Functional limitations were measured at wave 3. Both the LPA and theoretical approach resulted in 4 profiles (although there were qualitative differences), and both approaches differentiated covariate values in similar ways. Loglikelihood comparisons in Mplus showed that the LPA, compared to the theoretical approach, was more sensitive to detecting differences in functional limitations. Overall, the data-driven profiles differed from theory-based profiles, which may be substantively meaningful for understanding associations between life-course relationships and health. Practical implications include development of a measure for future research to apply these novel life-course social relationship groups, particularly with smaller samples not suitable for LPA.

